# Bitter Gourd Honey Ameliorates Hepatic and Renal Diabetic Complications on Type 2 Diabetes Rat Models by Antioxidant, Anti-Inflammatory, and Anti-Apoptotic Mechanisms

**DOI:** 10.3390/foods10112872

**Published:** 2021-11-20

**Authors:** Chandra Sekhar Arigela, Giribabu Nelli, Siew Hua Gan, Kuttulebbai Nainamohamed Salam Sirajudeen, Kumarathevan Krishnan, Nurhanan Abdul Rahman, Visweswara Rao Pasupuleti

**Affiliations:** 1 Faculty of Agro-Based Industry, Universiti Malaysia Kelantan, Campus Jeli, Kota Bharu 17600, Kelantan, Malaysia; chandrabiotech3@gmail.com (C.S.A.); thevan@umk.edu.my (K.K.); 2 Department of Physiology, Faculty of Medicine, University of Malaya, Kuala Lumpur 50603, Selangor, Malaysia; nelli.giribabu@um.edu.my; 3 School of Pharmacy, Monash University Malaysia, Jalan Lagoon Selatan, Bandar Sunway 47500, Selangor, Malaysia; Gan.siewhua@monash.edu; 4 Department of Basic Medical Sciences, Kulliyyah of Medicine, International Islamic University Malaysia (IIUM), Bandar Indera Mahkota, Kuantan 25200, Pahang, Malaysia; knssiraj@iium.edu.my; 5 Department of Biomedical Sciences and Therapeutics, Faculty of Medicine & Health Sciences, Universiti Malaysia Sabah, Kota Kinabalu 44800, Sabah, Malaysia; 6 Department of Biochemistry, Faculty of Medicine and Health Sciences, Abdurrab University, Pekanbaru 28291, Riau, Indonesia; 7 Centre for Excellence in Biomaterials Engineering (CoEBE), AIMST University, Bedong 08100, Kedah, Malaysia

**Keywords:** diabetes mellitus, oxidative stress, antioxidant enzymes, inflammation

## Abstract

Honey has several pharmacological effects, including anti-diabetic activity. However, the effectiveness of bitter gourd honey (BGH) in the treatment of diabetes mellitus (DM) is unknown. The aim of this study was to determine the antioxidant, anti-inflammatory, and anti-apoptotic properties of BGH on the kidney and liver of a streptozotocin-induced diabetes rat model. Methods: A single dose (nicotinamide 110 mg/kg, streptozotocin (STZ) 55 mg/kg, intraperitoneal (i.p.)) was used to induce DM in male rats. For 28 days, normal or diabetic rats were administered 1 g/kg/day and 2 g/kg/day of BGH orally. After the treatment, blood, liver, and kidney samples were collected and analysed for biochemical, histological, and molecular parameters. In addition, liquid chromatography–mass spectrometry (LC-MS) was used to identify the major bioactive components in BGH. Results: The administration of BGH to diabetic rats resulted in significant reductions in alanine transaminase (ALT),aspartate aminotransferase (AST), creatinine, and urea levels. Diabetic rats treated with BGH showed lesser pathophysiological alterations in the liver and kidney as compared to non-treated control rats. BGH-treated diabetic rats exhibited reduced levels of oxidative stress (MDA levels), inflammatory (MYD88, NFKB, p-NFKB, IKKβ), and apoptotic (caspase-3) markers, as well as higher levels of antioxidant enzymes (SOD, CAT, and GPx) in the liver and kidney. BGH contains many bioactive compounds that may have antioxidative stress, anti-inflammatory, and anti-apoptotic effects. Conclusion: BGH protected the liver and kidney in diabetic rats by reducing oxidative stress, inflammation, and apoptosis-induced damage. As a result, BGH can be used as a potential therapy to ameliorate diabetic complications.

## 1. Introduction

Human beings are undoubtedly exposed to extrinsic and intrinsic (oxidative, electrophilic) chemicals [1]. Constant stress from these chemicals disrupts animal cell homeostasis by altering cell lipids, proteins, and nucleic acids, resulting in chronic diseases such as diabetes mellitus, cancer, neurodegenerative diseases, cardiovascular disease, and diabetes mellitus (DM) [2,3]. In addition, type 2 diabetes mellitus (T2DM) is a complex hereditary and environmental disease. T2DM is defined by pathophysiological changes in pancreatic cells as well as increased peripheral (e.g., liver, kidney, skeletal muscle, and adipose) tissue reactive oxygen species (ROS) and inflammation, which can occur downstream of the insulin receptor (IR) and its signalling pathways, resulting in an inadequate response to insulin levels. This causes insulin resistance and chronic inflammation, which impair blood glucose control and contribute to the development of microvascular (nephropathy, liver cirrhosis, neuropathy, and retinopathy) and macrovascular (coronary heart disease, peripheral vascular disease, and stroke) complications. DM could be the leading cause of liver disease, with many hepatic complications in patients with diabetes associated with increased predominant liver problems, including liver and portal hypertension, non-alcoholic steatohepatitis (NASH), fibrosis, cirrhosis, liver carcinoma, and acute liver failure [4]. On the other side, liver failure is a major underlying cause of T2DM mortality, where cirrhosis accounted for 12.5% of all causes of death in one prospective cohort study [5]. Similarly, 20–30% of T2DM patients had renal impairment. Unfortunately, the combination of diabetes and chronic kidney disease (CKD) is often attributed to increasing morbidity and mortality [6,7]. As a consequence, it is essential to address the unmet medical need for diabetic complications. Diabetes and hyperglycaemia have been linked to increased intracellular ROS, which causes cell damage and inflammation. Hence, an approach to fight against both inflammation and oxidative injury will effectively minimise the risk of diabetic complications [8]. Eukaryotic cells are equipped with complicated protective mechanisms to protect against oxidative and electrophilic chemicals [9]. In the defence against cellular oxidative damage, antioxidant responsive element (ARE)-regulated gene expression plays a crucial role. Nuclear erythroid factor 2 (Nrf2) is one of the key ARE-binding transcription factors. The protein associated with the Nrf2 target gene has a broad range of cytoprotective properties, including antioxidation, anti-inflammatory, and detoxification. Multiple studies have shown that the Nrf2 pathway plays an important role in the reduction of oxidative stress [10].

For many years, natural products have evolved to have rich chemical diversity with extensive, versatile biological activities and remain as abundant and powerful resources for the development of therapeutic agents. Few researchers have investigated the crucial role of various natural polyphenols in disease prevention as well as in food and medicine [11,12]. At present, the discovery and optimization of leading molecules to therapeutically significant natural products have become a prominent strategy in contemporary research in medicinal chemistry. Insulin resistance and other symptoms of type 2 diabetes may be improved by some Nrf2 activators such as quercetin and resveratrol. Increasing insulin sensitivity by controlling Nrf2 levels is a potential avenue to treat type 2 diabetes [13,14]. Honey is a natural product with various pharmacological roles in both humans and animals. Researchers have found that honey may be used as an antioxidant to prevent oxidative damage to various organs, including the kidney, pancreases, liver, and heart [15], while recent studies have shown that honey plays a role in reducing oxidative stress-related cell death as well as the apoptosis of testicular germ cells [16]. According to previous studies, the antioxidant properties of Tualang honey helped diabetic rats with STZ-induced hyperglycaemia by reducing lipid peroxidation as well as enhancing superoxide dismutase (SOD) and glutathione peroxidase (GPx) activity [17]. Honey has anti-inflammatory properties in both acute and chronic inflammation, including arthritis [18]. Similarly, studies have shown that Tualang honey and Manuka honey and their constituents have anti-inflammatory properties [19]. Tualang honey was used to treat chemically-induced corneal damage in a rabbit, and the outcomes were equivalent to conventional therapy [20]. Recently identified Gelam honey can reduce inflammatory mediators such as TNF-α and COX-2 by suppressing NF-κB translocation to the nucleus and therefore limiting the activation of the NF-κB pathway [21]. To date, no studies have been reported that elucidate the precise mechanism of the protective effect of BGH on diabetics and its complications. In the present study, we set out to investigate the protective effects of BGH and further the underlying mechanism of BGH on inflammation, oxidative stress, and apoptosis in the pathophysiology of the liver and kidney in streptozotocin-induced diabetic rats.

## 2. Materials and Methods

### 2.1. Materials and Kits

The Wild Bitter Gourd Honey (BGH) was purchased from ECO BEE SHOP SDN BHD Johor, Malaysia. Streptozotocin and nicotinamide were purchased from Sigma-Aldrich (St. Louis, MO, USA).

### 2.2. Liquid Chromatography-Mass Spectroscopy (LC-MS) 

LCMS analysis was performed in accordance with the previously described method [22]. To identify the bioactive compounds in the BGH extracts, an LC-MS (Accurate Q-TOF mass Spectrometer) coupled to HPLC, equipped with a UV detector, is utilized. For the chromatographic separation of phenolic compounds, an ACQUITY UPLC HSS T3 column (100 mm × 2.1 mm × 1.8 μm) was used. The linear binary water gradient (formic acid 0.1%) and an acetonitrile (mobile phase B) gradient were used as the mobile phases A and B, respectively. During the run, the mobile process’s composition changed over the period: 1% B for 0 min; 1% B for 0.5 min; 35% B for 16.00 min; 100% B for 18.00 min; 1% B for 20.00 min with the injection volume of 1 μL and at a flow rate of 0.6 mL/min. Electrospray ionisation (ESI) parameters; both negative and positive ion modes; mass range of 50–1500 m/z; spray voltage 3 kV, gas temperature 5500 C, gas flow 800 L/h. As desolvation and cone gas, 99.5% nitrogen was used. Mass was analysed by using a TWIMS-QTOFMS analysis.

### 2.3. Molecular Docking

Molecular docking studies were performed using Auto Dock Vina, which was implemented in an open-source tool called PyRx 0.8 [23]. A grid was prepared around the active site of selected target proteins viz., α-amylase (PDB id: 4w93) [24] and PPAR-γ (PDB id: 4ema) [25], and docked with compounds to predict their binding energies and binding modes.

Redocking was performed to validate that the docking parameters given in the input file for the docking method are rational enough to recapitulate the ligand–protein complex intrinsic interactions. PDB Ids 4W93 and 4ema have 3d coordinates of α-amylase crystallised with montbretin A and PPAR-γ crystallised with rosiglitazone, respectively. The ligands were removed and docked back into their respective active sites. 

### 2.4. Animal Studies

This study was conducted out on eight-week-old male Sprague–Dawley (SD) rats weighing about 180 ± 220 g. Animals were bred and raised at the University of Malaya Experimental Unit’s animal house facility. 

The Institutional Animal Care and Use Committee (IACUC) of the University of Malaya approved all investigations, and they were also accredited by the Association for Assessment and Accreditation of Laboratory Animal Care International (AAALAC). The rats were housed in a properly ventilated atmosphere with a natural light/dark cycle (12 h light/dark cycle), temperature of 24 ± 1 °C, 50–70% humidity. A standard laboratory pellet diet and distilled water ad libitum were given to rats during the experiment.

### 2.5. Induction of Diabetes in Animal Model

To induce T2DM in male adult rats, streptozotocin (catalogue number: S0130; Sigma-Aldrich, St. Louis, MO, USA) and nicotinamide (NA) were administered. Rats were allowed to fast overnight prior to streptozotocin (STZ) administration. Adult rats were given NA—110 mg/kg dissolved in saline intraperitoneally 15 min before receiving STZ—55 mg/kg i.p. i.e., STZ (product code S0130-1G, Sigma Aldrich, St. Louis, MO, USA) dissolved in 0.1 M citrate buffer (pH 4.5) before use [26]. To prevent hypoglycaemia, all rats have access to drink 5% glucose for 6 h. After 72 h, fasting blood glucose levels (FBG) were measured by collecting a drop of blood from the tail vein. Rats were considered as diabetic with FBG > 12 mmol/L.

### 2.6. Experimental Design

A total of 56 rats were divided randomly into seven groups, with six rats in each group. Treatment was initiated on day 4th after DM conformation. 

Group 1—Normal, non-diabetic control (NC) treated with vehicle

Group 2—Normal, non-diabetic treated with BGH at 1 g/kg/day (NC + H1)

Group 3—Normal, non-diabetic treated with BGH at 2 g/kg/day (NC + H2)

Group 4—Diabetic control (DC) treated with vehicle

Group 5—Diabetic, treated with BGH at 1 g/kg/day (DC + H1)

Group 6—Diabetic, treated with BGH at 2 g/kg/day (DC + H2)

Group 7—Diabetic, treated with 2 mg/kg/day glibenclamide (DC + GB).

BGH and glibenclamide were administered orally for 28 consecutive days with an oral gauge tube. Saline (0.5 mL/kg) was used as a vehicle for control and diabetic rats during the treatment time. The doses of BGH (1 and 2 g/kg/b.w./day) were chosen based on recent research studies involving the administration of honey to diabetic rats [26,27]. Glibenclamide (2 mg/kg/day) was used in this study as a positive control [28]. The animals were sacrificed by inhaling carbon dioxide (CO2), and blood was collected from the heart into non-heparinized tubes. The blood (3 mL) was allowed to coagulate at room temperature for 2 h before being centrifuged at 1100× *g* for 15 min at 4 °C, and serum was separated and deposited at −20 °C. The liver and kidney were collected and examined for morphological and molecular changes.

### 2.7. Determination of Serum Hepatic and Renal Markers

Serum urea, creatinine, aspartate aminotransferase (ALT), and alanine aminotransferase (AST) were determined by using Siemens-ADVIA 2400 (Siemens Healthcare Diagnostics Inc., Deerfield, IL, USA) according to the manufacturer’s protocol.

### 2.8. Masson’s Trichome and Periodic acid Schiff (PAS) Staining of Liver and Kidney Tissue

Excised liver and kidney tissue were fixed with 4% formalin and embedded in paraffin wax. Paraffin wax-embedded tissues were sectioned at 5 μm using a semi-automated microtome (RM2155; Leica Micro-systems, Hessen, Germany). Tissue sections were mounted on glass slides with a hot plate. Sections were deparaffinised by immersing in xylene and subsequently hydrated using two changes of 100% of ethanol, followed by 95%, 80%, and tissues sections were stained with Masson’s Trichrome kit (catalogue number: ab150686, Abcam, Cambridge, UK). In Masson’s Trichrome stained slides, collagen and other extracellular matrix (ECM) components were examined. Fibrosis was stained blue, whereas parenchyma was stained red.

Periodic acid Schiff (PAS) (catalogue number: Ab150680, Abcam, Cambridge, UK) staining was used to investigate mucopolysaccharides and fat accumulation in the tissue sections in accordance with the manufacturer protocol. The tissue sections were incubated for 10 min with 0.5% periodic acid, followed by 10–20 min with the Schiff reagent, and then rinsed with water for 5 min. After various dehydration steps with an increased percentage of ethanol (75%, 95%, and 100%), they were cleared with xylene and mounted using mounting media. Slides were observed using a phase-contrast microscope with an attached photographic unit (Nikon H600L), Nikon DS camera controller DS U2, version 4.4, Tokyo, Japan) for the assessment of microscopic variation in the harvested tissues.

### 2.9. Determining the Levels of Antioxidant Enzyme and Oxidative Stress Marker in Liver and Kidney Homogenates

Liver and kidney tissue homogenates were prepared using a glass Teflon homogenizer (Hei-dolph Silent Crusher M, Schwabach, Germany) in a phosphate buffer (0.1 M, pH 7.4). The cytosolic fractions were prepared and used to quantify endogenous antioxidant enzymes and lipid peroxidation (LPO). The LPO was determined by the reaction of thiobarbituric acid (TBA) to malondialdehyde (MDA), where the latter was a compound formed by the membrane lipid peroxidation by the Esterbauer and Cheeseman method [29]. This level of LPO was expressed in the form of millimoles of MDA formed/g wet tissue weight. The activity of SOD was assayed by the Misra et al. method [30]. The study method involves the inhibition of epinephrine autoxidation through adrenochrome in the presence of the SOD in the alkaline medium (pH 10.2). The rate of SOD activity was expressed as the amount of enzyme that prevents epinephrine oxidation by 50% and is equivalent to U/mg of protein. The catalyse activity was assayed according to the method described by Chance et al. [31]. The activity of CAT enzymes was based on the decomposition of hydrogen peroxide (H_2_O_2_) and reported as mol of H_2_O_2_ metabolised/mg protein/min. The activity of GPx was determined by using the Rotruck and Pope method [32], which was expressed as μmol of GSH consumed/mg protein/min.

### 2.10. Immunohistochemistry and Immunofluorescence

The 5 μm sections were deparaffinized and rehydrated through ethanol (100% for 10 min, and 95, 90, 80, and 70% for 5 min each). They were finally washed with PBS and distilled water for 5 min. Antigen retrieval was conducted by heating the sections in a citrate buffer of 0.01 M, pH 6.0 at 95° C for 15 min, and cooled temperature. Endogenous peroxides were blocked by using 3% hydrogen peroxide (catalogue number: ab64218 Abcam, Cambridge, UK) for 30 min at room temperatures and washed with PBS. Sections were blocked for non-specific binding with Blocking One Histo (catalogue number: 06349-64, Nacalai tesque, Tokyo, Japan) for 60 min. The sections were incubated with primary antibodies, i.e., MYD88 (catalogue number: NB100-56698; from Novus Biologicals, CA, USA), IKKβ (cat.no. ab59195 Abcam, Cambridge, UK), and caspase 3 (catalogue number: 9662 Cell Signalling Technology, Danvers, MA, USA) at a 1:1000 ratio overnight at 4 °C, and the following day, slides were washed with PBS, which was accompanied by respective secondary antibodies anti-mouse, anti-rabbit (catalogue number:sc-516102; catalogue number: sc-2357; Santa Cruz Biotechnology, Inc., Santa Cruz, CA, USA) incubation for 90 min at 37 °C. Then, slides were washed with PBS twice, each for 5 min, and sections were visualised by using diaminnobenzidine-tetra-hydrochloride (DAB kit; catalogue number: ab64238 Abcam, Cambridge, UK), counterstained with haematoxylin followed by being dehydrated, cleared, and coverslipped, and sections were imaged using phase-contrast microscope (Nikon H600L, Nikon DS camera control unit DS-U2, version 4.4, Tokyo, Japan). The intensity of immunohistochemistry was quantified via Image J software (NIH, Bethesda, MD, USA) and expressed as pixel luminosity.

Immunofluorescence was performed by using the VectaFluorTM Duet Double Labeling Kit, DyLight^®^ 594 Anti-Rabbit IgG, DyLight^®^ 488 Anti-Mouse IgG (catalogue number: DK-8828, Vector Laboratories, Inc., Burlingame, CA, USA). Sections were blocked with 2.5% horse serum to avoid nonspecific binding. After that, the first primary antibodies—i.e., HO-1 (catalogue number: NBP1-97507 Novus Biologicals, Centennial, CO, USA) and NF-κB (catalogue number: 956; Cell Signaling Technology, Danvers, MA, USA)—were incubated overnight at 4 °C, washed with PBS twice for 5 min each, and incubated for 2 h with the second primary antibodies Nrf2 (catalogue number: NBP1-32822; Novus Biologicals, Centennial, CO, USA) and P-NF-B (catalogue number: 3033; Cell Signaling Technology, Danvers, MA, USA), which was followed by PBS washing. Then, the sections were incubated with Vecta FluorTM Duet (Vector Laboratories, Inc., Burlingame, CA, USA) Reagent for 60 min. Finally, the sections were washed twice with PBS and mounted with vectashield antifade Mounting media (Vector Laboratories, Inc., Burlingame, CA, USA), after which we observed immunological changes under a confocal laser scanning microscope (Leica DM400B; Leica Microsystems, Wetzlar, Germany). The fluorescence intensity was quantified via Image J software (NIH, Bethesda, MD, USA) and expressed as the mean of fluorescence intensity.

### 2.11. Statistical Analysis 

The statistical analysis was carried out using the statistical software Graph Pad Prism Version 5 (Graph Pad Software Inc., San Diego, CA, USA). The results were shown as mean ± S.E.M. One-way analysis of variance (ANOVA) was used to compare data among the groups; then, Tukey’s multiple comparison method was used to compare means between groups. *p*-value < 0.05 was considered significant.

## 3. Results

### 3.1. Identification of Polyphenols in BGH by LC-ESI-MS/MS

**Figure 1:**
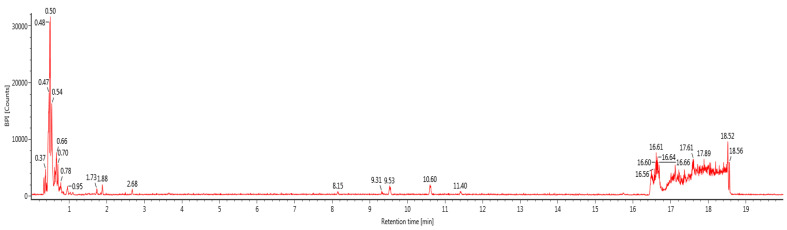
LC-MS chromatogram of BGH sample spectral data is listed in Table 1. The *x*-axis shows retention time in minutes; the *y*-axis shows the intensity.

Bioactive compounds (polyphenols) were identified in BGH, based on the optimization conditions of LC-ESI-MS/MS by searching information in the standard library (e.g., peak retention times, UV range, [M-H] (m^2^) and ESI-MS/MS data). All compounds were identified with a 0.4 to 7.62 retention time (RT) range in the total ion chromatogram. The identified compounds were listed in Table 1 and the chromatogram is shown in Figure 1. 

### 3.2. Molecular Docking against Detected Compounds in LCMS

All selected compounds showed good binding energies against the active sites of target proteins (Table 2). When compared to other compounds, shancilin and iridin had higher binding energies against amylase, while shancilin, lamiophlomiol_A, asperuloside, and coumaric acid had higher binding energies against PPAR-gama.

**Figure 2:**
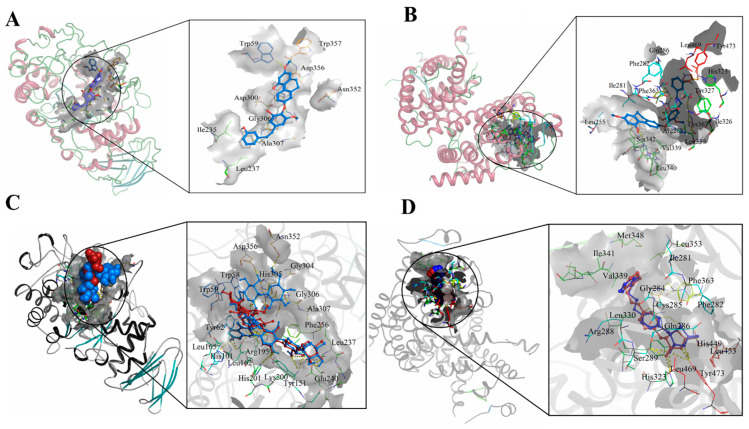
(**A**) Docking image Amylase—shancilin, (**B**) Docking image PPAR-γ—shancilin, (**C**) Redocked image amylase—Montbretin, (**D**) Redocked image PPAR-γ—rosiglitazone.

Among all the selected compounds, shancilin showed better binding affinities against two selected targets. The binding mode is shown in Figure 2A,B. Shancilin and iridin form a hydrogen bond with Trp59 of amylase. Shancilin forms a hydrogen bond with His323. Redocked Montbretin A showed hydrogen bonding with His101, Tyr151, Arg195, Asp197, Lys200, His201, Glu233, Glu240, Asp356, and Asn352 of αamylase with a binding score of 9.1 kcal/mol, while the PDB structure shows hydrogen bonding with His101, Arg195, Asp197, Lys200, His201, Glu233, Ile235, and Glu240 (Figure 2C Red-pdb, blue-redock). Redocked rosiglitazone showed hydrogen bonding with His323 and Tyr473 of PPAR-γ with a docking score of -8.4 kcal/mol, while its structure deposited in the PDB showed hydrogen bonding with His449, Tyr473, and His323 (Figure 2D Red-pdb, blue-redock).

### 3.3. Effect of BGH on Liver, Kidney Functional Markers

**Figure 3:**
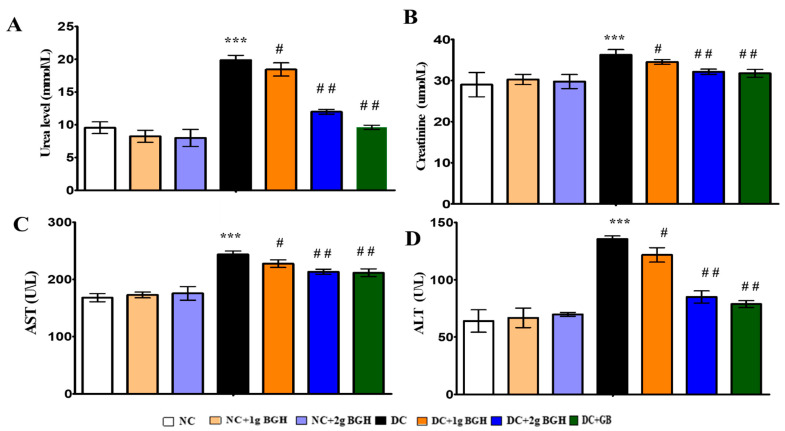
Bar charts showing the effect of BGH on liver and kidney functional markers: (**A**) Urea levels, (**B**) Creatine levels, (**C**) Aspartate aminotransferase (AST) levels, (**D**) Alanine aminotransferase (ALT) levels. The results were collected from six separate rats within every treatment group and were reported as a mean ± S.E.M. *** *p* < 0.001 vs. NC; # *p* < 0.05; ## *p* < 0.01 vs. DC. NC: Normal control; NC+1g BGH: Non-diabetic rats that received 1 g/kg/day BGH; NC + 2 g BGH: Non-diabetic rats that received 2 g/kg/day BGH; DC: Diabetic control; DC + 1 g BGH: Diabetic rats that received 1 g/kg/day BGH; DC + 2 g BGH: Diabetic rats that received 2 g/kg/day BGH; DC + GB: Diabetic rats that received 2 mg/kg/day glibenclamide.

The serum levels of urea, creatinine, AST, and ALT were measured to assess renal and hepatic function and injury. Diabetic rats were shown to increase urea (Figure 3A), serum creatine (Figure 3B), AST (Figure 3C), and ALT (Figure 3D) significantly (*p* < 0.05) compared to non-diabetic rats. Treatment with 2 g/kg/day BGH and glibenclamide significantly (*p* < 0.01) reduced serum levels of renal function and hepatic function markers in diabetic rats. No significant changes were observed with oral BGH supplementation in non-diabetic control rats. 

### 3.4. Effect of BGH on Histopathological Changes in Diabetic Rats

**Figure 4:**
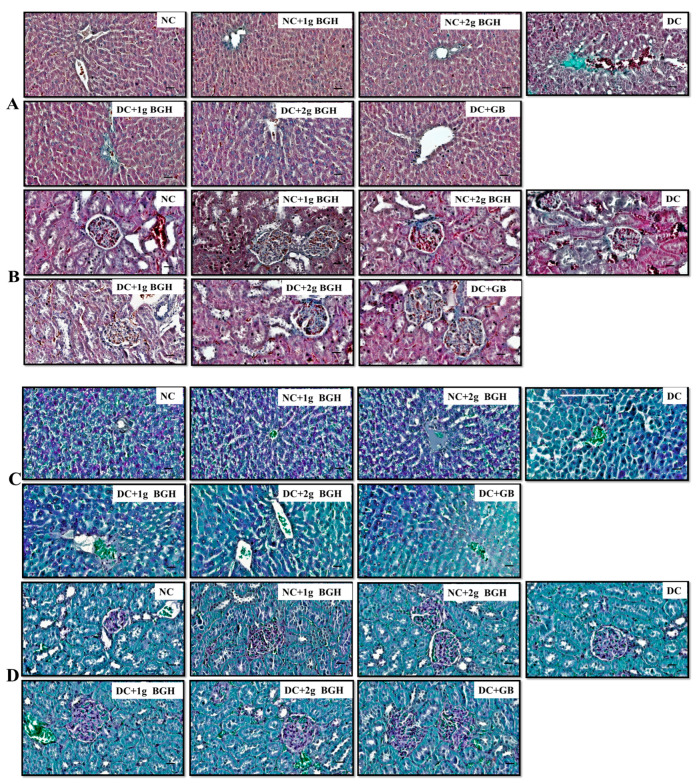
BGH effect on histopathological differences in the appearance of hepatic cells (**A**) and renal cells (**B**) in various experimental groups, which were stained with Masson trichrome. BGH effect on histopathological differences in the appearance of l hepatic cells (**C**) and renal cells (**D**) in various experimental groups, which were stained with Periodic acid—Schiff. Scale bar = 100 µm. NC: Normal control; NC + 1 g BGH: Non-diabetic rats that received 1 g/kg/day BGH; NC + 2 g BGH: Non-diabetic rats that received 2 g/kg/day BGH; DC: Diabetic control; DC + 1 g BGH: Diabetic rats that received 1 g/kg/day BGH; DC + 2 g BGH: Diabetic rats that received 2 g/kg/day BGH; DC + GB: Diabetic rats that received 2 mg/kg/day glibenclamide.

Diabetic rats were prone to developing acute lipid metabolism disorders, and high-level glucose constantly induced liver dysfunction, leading to liver fibrosis. The therapeutic effect of BGH on liver fibrosis is shown in Figure 4A,C. Masson’s trichrome and Periodic acid–Schiff are histological techniques used to demonstrate collagen and glycogen presence, respectively. The diabetic control rat liver shows evident connective tissue hyperplasia and increased ECM content as well as collagen accumulation in the lobules, resulting in large fibrous septa and pseudo-lobule formation compared with that of non-diabetic rats. After treatment with 1 g/kg/day or 2 g/kg/day BGH and glibenclamide, we noticed fewer changes in liver fibrosis. The PAS staining assessment revealed a significant amount of glycogen storage in non-diabetic hepatocytes. In contrast, the intracellular glycogen depository was reduced in the diabetic group, and the glycogen levels were restored with 1 g/kg/day or 2 g/kg/day BGH and glibenclamide treatment in diabetic rats. Lesser liver fibrosis and glycogen depletion changes were observed by oral treatment of 1 g/kg/day or 2 g/kg/day BGH in non-diabetic control rats.

Higher blood glucose levels in each kidney over time destroy millions of tiny philtres, leading to renal failure, as shown in Figure 4B,D. The kidney of diabetic control rats has shown increased collagen deposition as well as the degeneration of cortical tubules and tubular dilation as compared with that of non-diabetic rats. Treatment of diabetic rats with 1 g/kg/day or 2 g/kg/day BGH and glibenclamide reduced collagen within the interstitium, and there was no apparent tubular pathology. In diabetic rats, glomerulosclerosis was distinguished by glomerular basement membrane thickness and glomerular hypertrophy, and an accumulation of mesangial matrix was observed as compared with the treatment of diabetic rats with 1 g/kg/day or 2 g/kg/day BGH and glibenclamide. Fewer changes were observed in kidney pathophysiology by the oral intubation of 1 g/kg /day or 2 g/kg/day BGH in non-diabetic control rats.

### 3.5. Effect of BGH on Antioxidant Enzyme in Liver and Kidney Homogenates

Lipid peroxidation product MDA formation was shown in different groups. The MDA levels were significantly (*p* < 0.01) higher in diabetic control rats compared to non-diabetic rats. When diabetic rats were given 1 g/kg/day or 2 g/kg/day BGH and glibenclamide, MDA levels in the liver (Table 3) and kidney (Table 4) homogenates were significantly (*p* < 0.05) lower than in diabetic control rats. There were no significant changes in MDA levels in non-diabetic rats, with 1 g/kg/day or 2 g/kg/day BGH.

The tissue antioxidant functions were disrupted due to higher oxidative stress in diabetic rats, as shown in the results. SOD activity levels were reduced significantly (*p* < 0.001) in diabetic rats when compared to non-diabetic rats. Treatment of diabetic rats with 1 g/kg/day and 2 g/kg/day BGH and glibenclamide resulted in significantly (*p* < 0.05) increased activity levels of SOD, CAT, and GPx in liver (Table 3) and kidney (Table 4) homogenates compared to that of diabetic controls (*p* < 0.05). No significant changes in SOD, CAT, and GPX activity levels were observed by the oral intubation of 1 g/kg/day or 2 g/kg/day BGH in non-diabetic control rats.

### 3.6. Effect of BGH on the Expression of Transcription Factor Nrf2 and NOQ1 in the Hepatocyte and Renal Tissues of Diabetic Rats

**Figure 5:**
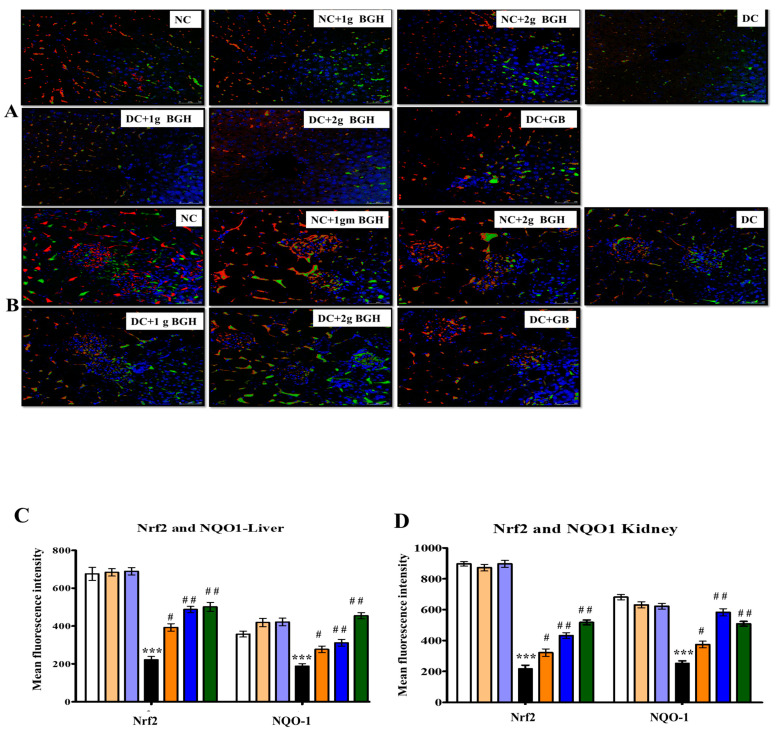
Representative confocal images of antioxidant markers Nrf2 (red) and NOQ1 (green) double immunofluorescence staining in the liver (**A**) and kidney (**B**) samples from BGH-treated rats. Quantitative analyses of immunofluorescence signals in the liver (**C**) and kidney tissues (**D**). The results were collected from six separate rats within every treatment group and were reported as a mean ± S.E.M. *** *p* < 0.001 vs. NC; # *p* < 0.05; ## *p* < 0.01 vs. DC. S Scale bar = 100 µm. NC: Normal control; NC+1g BGH: Non-diabetic rats that received 1 g/kg/day BGH; NC+2g BGH: Non-diabetic rats that received 2 g/kg/day BGH; DC: Diabetic control; DC+1g BGH: Diabetic rats that received 1 g/kg/day BGH; DC + 2g BGH: Diabetic rats that received 2 g/kg/day BGH; DC+GB: Diabetic rats that received 2 mg/kg/day glibenclamide.

Nrf2 is a transcription factor that regulates the expression of many antioxidants and defensive proteins that are redox-sensitive. By using the immunofluorescence technique, Nrf2 and NQO-1 expression levels were determined in the liver (Figure 5A) and kidney (Figure 5B) of non-diabetic and diabetic rats treated with BGH. Immunofluorescence images showed that Nrf2 and NQO-1 proteins were expressed comparatively lower in the liver and kidney of diabetic rats compared with non-diabetic rats. In contrast, the treatment of diabetic rats with 2 g/kg/day BGH and glibenclamide showed a higher distribution of Nrf2 and NQO-1 levels in the liver and kidney. In non-diabetic rats, the distribution of Nrf2 and NQO-1 were higher when either untreated or treated with 1 g/kg/day or 2 g/kg/day BGH.

### 3.7. Effect of BGH on the Expression of Inflammation Markers, MyD88–IKKB–NF-κB in the Hepatocyte and Renal Tissues of Diabetic Rats

**Figure 6:**
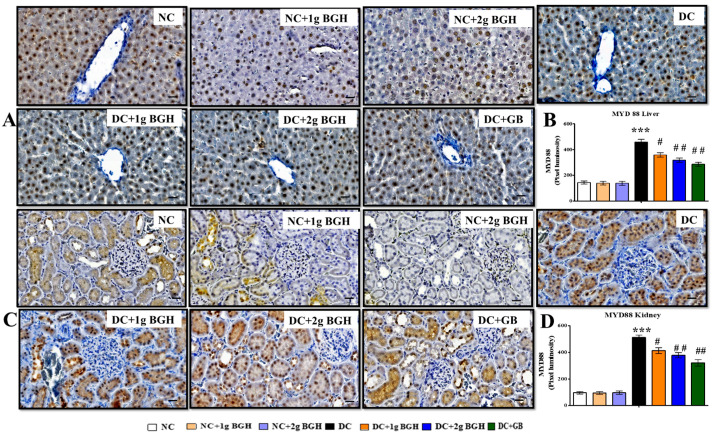
Effect of BGH on the expression of inflammation markers MyD88. Representing immunoperoxidase images showing the distribution of MYD88 in the hepatic cells (**A**) and renal cells (**C**). Bar chart showing the mean intensity of dark brown staining of MyD88 expression in hepatic cells (**B**) and renal cells (**D**). Data were obtained from six different rats in each treatment group and expressed as mean ± S.E.M. *** *p* < 0.001 vs. NC; # *p* < 0.05; ## *p* < 0.01; vs. DC. Bar scale = 100 μm. 40× Magnification. NC: Normal control; NC+1g BGH: Non-diabetic rats that received 1 g/kg/day BGH; NC + 2 g BGH: Non-diabetic rats that received 2 g/kg/day BGH; DC: Diabetic control; DC + 1 g BGH: Diabetic rats that received 1 g/kg/day BGH; DC + 2 g BGH: Diabetic rats that received 2 g/kg/day BGH; DC+GB: Diabetic rats that received 2 mg/kg/day glibenclamide.

**Figure 7:**
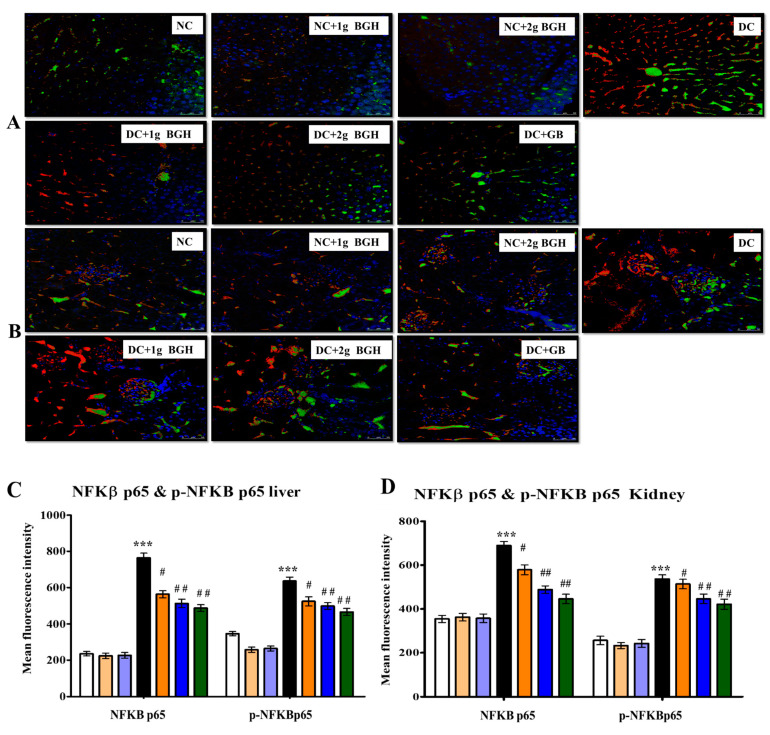
Representative confocal images of inflammatory markers p-NF-κB (red) and NF-κB (green) double immunofluorescence staining in the liver (**A**) and kidney (**B**) samples from BGH-treated rats. Quantitative analyses of immunofluorescence signals in the liver (**C**) and kidney sections (**D**). Data were obtained from six different rats in each treatment group and expressed as mean ± S.E.M. *** *p* < 0.001 vs. NC; # *p* < 0.05; ## *p* < 0.01 vs. DC. S Scale bar = 100 µm. NC: Normal control; NC+1g BGH: Non-diabetic rats that received 1 g/kg/day BGH; NC + 2 g BGH: Non-diabetic rats that received 2 g/kg/day BGH; DC: Diabetic control; DC + 1 g BGH: Diabetic rats that received 1 g/kg/day BGH; DC + 2 g BGH: Diabetic rats that received 2 g/kg/day BGH; DC + GB: Diabetic rats that received 2 mg/kg/day glibenclamide.

**Figure 8:**
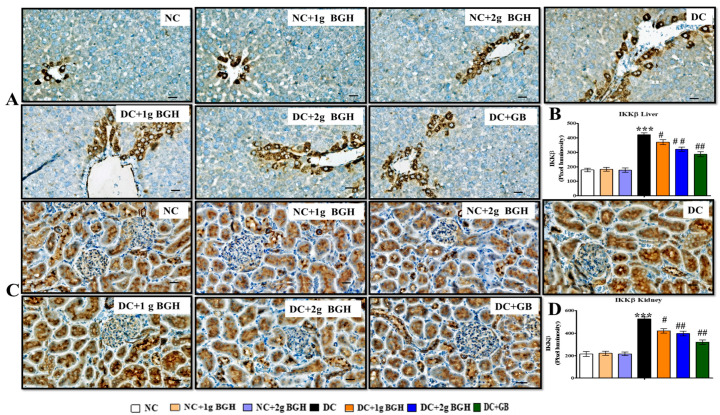
Effect of BGH on the expression of inflammation markers IKKβ. Representing immunoperoxidase images showing the distribution of IKKβ in the hepatic cells (**A**) and renal cells (**C**). Bar chart showing mean intensity of dark brown staining of IKKβ expression in hepatic cells (**B**) and renal cells (**D**). The results were collected from six separate rats within every treatment group and were reported as a mean ± S.E.M. *** *p* < 0.001 vs. NC; # *p* < 0.05; ## *p* < 0.01 vs. DC. Bar scale = 100 μm. 40× Magnification. NC: Normal control; NC + 1 g BGH: Non-diabetic rats that received 1 g/kg/day BGH; NC + 2 g BGH: Non-diabetic rats that received 2 g/kg/day BGH; DC: Diabetic control; DC + 1 g BGH: Diabetic rats that received 1 g/kg/day BGH; DC + 2 g BGH: Diabetic rats that received 2 g/kg/day BGH; DC + GB: Diabetic rats that received 2 mg/kg/day Glibenclamide.

We investigated whether BGH can prevent hepatic fibrosis and tubulointerstitial fibrosis by downregulating the innate immune MyD88–NF-κB–IKKβ pathway. The inflammatory markers show expression levels i.e., MyD88 (Figure 6), NF-κB p65 (Figure 7), p-NF-κB p65 (Figure 7), and IKKβ (Figure 8) in hepatic and renal tissues of rats receiving a different treatment, respectively. A lower distribution of inflammatory markers such as MyD88, IKKβ, and NF-κB p65 was observed in hepatic and renal tissues in non-diabetic rats. In contrast, a higher distribution of inflammatory marker levels in the cytoplasm and hepatocyte membranes of Kupffer cells and cortical tubular epithelial cells in some glomerular cells was observed in diabetic rats, and there was a lower expression in 1 g/kg/day or 2 g/kg/day BGH and glibenclamide-treated diabetic rats. In non-diabetic rats, expression levels of inflammatory markers were low, either untreated or treated with 1 g/kg/day or 2 g/kg/day BGH.

### 3.8. Effect of BGH on the Expression of Apoptotic Marker in the Renal and Hepatocyte Tissues of Diabetic Rats

**Figure 9:**
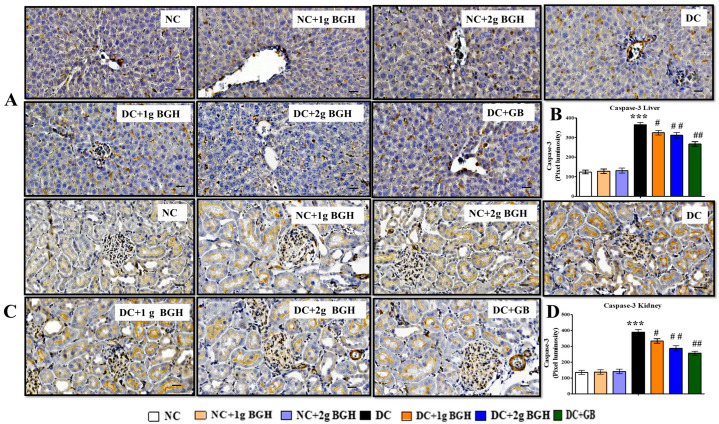
Effect of BGH on the expression of apoptosis marker caspase-3. Representing immunoperoxidase images showing the distribution of caspase-3 in the hepatic (**A**) and renal cells (**C**). Dark brown staining shows the locations of delivery. Bar chart showing mean intensity of dark brown staining of caspase-3 expression in hepatic cells (**B**) and renal cells (**D**). The results were collected from six separate rats within every treatment group and were reported as a mean ± S.E.M. *** *p* < 0.001 vs. NC; # *p* < 0.05; ## *p* < 0.01 vs. DC. Bar scale = 100 μm. 40× Magnification. NC: Normal control; NC + 1 g BGH: Non-diabetic rats that received 1 g/kg/day BGH; NC + 2 g BGH: Non-diabetic rats that received 2 g/kg/day BGH; DC: Diabetic control; DC + 1 g BGH: Diabetic rats that received 1 g/kg/day BGH; DC + 2 g BGH: Diabetic rats that received 2 g/kg/day BGH; DC + GB: Diabetic rats that received 2 mg/kg/day glibenclamide.

The immunostaining results in untreated diabetic rats showed a higher distribution of caspase 3 in the liver (Figure 9A) and kidney (Figure 9C) than in normal, non-diabetic rats. The treatment of diabetic rats with BGH at both doses (1 and 2 g/kg/day) as well as glibenclamide resulted in a lower expression of caspase 3 in the liver and renal tissues.

## 4. Discussion

Diabetes is distinguished by three significant metabolic abnormalities, including hyperglycaemia, hyperlipidaemia, and inflammation, which can induce the generation of reactive oxygen species (ROS). ROS were identified as significant drivers of diabetic complications such as diabetic nephropathy, non-alcoholic fatty liver disease (NAFLD), and cardiomyopathy [33,34,35,36]. T2DM can be induced by STZ, which involves damage to beta cells of pancreas by the alkylation of DNA (nitrosourea moiety). The STZ is a well-known donor of nitrogen oxide (NO); it is also responsible for the destruction of pancreatic β-cells and the modifications mentioned above, and it causes severe changes in blood glucose and insulin concentrations [37]. 

A high level of glucose promotes the formation of free radicals. As a result, it contributes to a lack of equilibrium between ROS and antioxidant protection. Polyphenols and flavonoids present in honey are powerful antioxidants due to their hydrogen-donating hydroxyl group and ability to donate electrons to prevent the production of free radicals [38,39,40]. This study was to elucidate the effect of STZ on pancreas beta cells and how BGH controls diabetes mellitus and its related symptoms such as oxidative stress, inflammatory, and hyperlipidaemia conditions. We found that BGH has strong antioxidant activity. This may be attributed to the polyphenols present in the BGH. For example, gallocatechin has been shown to possess antioxidant activity [41]. It has been shown that iridin has anti-inflammatory properties by preventing lipopolysaccharide-induced inflammatory responses of macrophages [42]. Phenolic compounds (Table 1) such coumaric acid and vanillic acid, among others, were identified in BGH using LCMS analysis, and wildflower honey and thyme honey supported these observations [43]. 

Our molecular docking studies showed that shancilin and iridin have greater binding energies against α-amylase and also against PPAR-γ when compared to other compounds. There is a possibility that these active constituents present in BGH may be binding to α-amylase and PPAR-γ and exerting the anti-diabetic mechanisms. It is known that amylase helps to downregulate GLUT2 and inhibits gut glucose absorption, and it possibly reduces insulin resistance in T2D animal models [44]. It is known that thiazolidinediones, a class of PPAR-γ agonists, have been shown to modulate gene expression involved in the control of glucose, lipid, and protein metabolism, therefore synergizing insulin action and increasing glucose uptake in insulin-sensitive tissues such as skeletal muscle and adipose tissue while reducing hepatic glucose synthesis [45]. It is suggested that BGH components may modulate pancreatic α-amylase and PPAR-γ, hence reducing diabetes complications in diabetic rats treated with BGH. 

Honey contains high fructose, which slows gastric emptying and reduces plasma glucose levels by stimulating glucokinase in the liver and β-cells to induce insulin production [46,47,48]. Consequently, the fructose, polyphenols, and flavonoids present in honey suppress hyperglycaemia-induced hepatic stress in diabetic rats.

ROS can directly oxidise and alter DNA, proteins, macromolecules, and lipids directly, as well as indirectly cause tissue damage by triggering a various number of cellular stress-sensitive pathways, hexosamines, protein kinases, and other enzymes. Serum urea and creatinine levels were measured to determine the renal damage caused by diabetes. In our study, 2 g/kg/day BGH significantly reduced renal damage, which was nearly identical to the result of glibenclamide treatment. Rats were fed with Tualang honey and showed similar results [17,49]. The AST and ALT levels are viewed as reliable markers of hepatocellular damage and can be used to assess the severity of liver damage. Intubation of 2 g/kg/day BGH significantly reduced the liver damage, which was almost similar to the effect of glibenclamide. Similar results were observed in rats treated with Tualang, Gleam, and Apica honey [50,51,52]. The increased urea and creatinine levels in the serum indicates impaired renal function [53]. The urea and creatinine levels were significantly reduced with BGH treatment. These results were supported by earlier work on the protective effect of Tulkarm honey on kidney function in rats; oral supplementation of Royal jelly honey reduced creatinine, urea, and uric acid levels in rats with cisplatin-induced damage in liver and kidney [54,55].

According to Simmons [56], mitochondrial dysfunction and oxidative stress play an significant role in the pathogenesis of T2D. In DM, lipid peroxidation and protein oxidation are commonly used as biomarkers to evaluate oxidative stress levels. Cells were evolved to have various antioxidant mechanisms, including antioxidant enzymes, i.e., SOD, CAT, and GPx, in response to excessive ROS and oxidative stress during metabolism. SOD catalyses the hydrolysis of superoxide radicals into oxygen and H_2_O_2_ [57]. In this preceding stage, hydrogen peroxide is also a toxic metabolite and is further eliminated by another catalase antioxidant enzyme; it hydrolyses H_2_O_2_ into water and oxygen [58]. Another antioxidant enzyme, GPx, also helps to eliminate H_2_O_2_ by converting it to water and oxygen. In the current study, the lipid peroxidation product MDA levels were reduced in the liver and kidney, while SOD, CAT, and GPx levels significantly increased with BGH treatment. Similar results were observed in previous studies, where Tualang honey treatment reduced elevated MDA and increased SOD, CAT, and GPX levels [17,59].

In diabetic rats, the MDA levels were significantly increased, and lower antioxidant defences were observed. The redox-sensitive Nrf2 protein was also significantly reduced in the liver and kidney of diabetic rats; this might be due to increased ROS and oxidative stress. Several studies have shown the significance of Nrf2 in the reduction of chronic inflammation as well as therapeutic activity [60,61]. Recent evidence has explored Nrf2 activation to reduce oxidative stress and strengthen antioxidant defences in the brain, kidney, liver, and endothelial cells [62,63,64,65,66]. 

The BGH-induced changes in the expression levels of Nrf2 in the liver and kidney of diabetic rats were observed. As a consequence of this Nrf2 activation, STZ-induced diabetic rats treated with BGH had shown lower lipid peroxidation and improved antioxidant defences. Interestingly, BGH improved Nrf2 expression in normal rats. As a result, it is proposed that daily BGH supplementation stimulates the endogenous antioxidant system. Furthermore, BGH anti-inflammatory effect may be linked to its ability to upregulate Nrf2. These pharmacological interventions have been further confirmed in the liver and kidney histologic sections, where BGH-treated diabetic rats attenuated the invasion of inflammatory leukocytes and prevented tissue damage. Similarly, Stingless bee honey had been shown to modulate Nrf2 signalling and reduce oxidative stress in chronic subclinical systemic inflammation (CSSI), and it was found that Nrf2 expression was upregulated by honey supplementation in hypertensive rats and resulted in a reduction of renal oxidative stress [67,68].

The NF-κB signalling pathway activation plays a vital role in the regulation of inflammation, which is related to hyperglycaemia, hyperlipidaemia, and inflammation in the liver and kidney. TNF-α, IIKβ, IL-6, and IL-1 as well as other chronic inflammatory cytokines are produced, when ROS triggers NF-κB. In addition, ROS-activated p38 MAPK [69] regulates TNF-α and IL-1β production [70]. Hence, the generation of inflammatory cytokines will be reduced by attenuating NF-κB and p38 MAPK signalling. Interestingly, our findings showed that oral 2 g/kg/day BGH supplementation significantly decreased the expression of both NF-κB, MYD88, and IKKβ in the liver and kidney of treated diabetic rats. These findings are supported by earlier works, where honey and its constituents to have anti-inflammatory properties by modulating NF-κB and LPS-induced pro-inflammatory cytokines in animal models [71,72,73,74,75].

Honey supplementation has been shown to exert neuroprotective effects by rescuing hippocampus cell death in STZ-induced diabetic rats [76]. Taulang honey has been reported to ameliorate neuroinflammation and reduce caspase-3 in a neuro-disease model of rats [77]. These studies indicate that honey has anti-apoptosis activity and reduces caspase-3. Similar results were observed in our study; caspase-3 levels were reduced in the liver and kidney of diabetic rats treated with BGH, indicating that BGH treatment decreased inflammation and reduced apoptosis. These results collectively indicate that BGH supplementation reduces diabetic complications by multiple mechanisms. Our study sheds light on the BGH and confirms that BGH has protective effects and rescues hepatic and renal tissues from diabetic complications through antioxidant, anti-inflammatory, and anti-apoptosis mechanisms. Furthermore, BGH can act as a naturally rich source of active molecules that can help novel drug discovery for various medically important diabetic ailments.

## 5. Conclusions

BGH supplementation reduced diabetic complications in the hepatic and renal tissues in diabetic rat models. BGH exerts its benefits by downregulating inflammation-associated markers MyD88, NF-κB p65, p-NF-κB p65, and IKKβ, reducing apoptosis and fibrotic collagen deposition while upregulating antioxidant mechanisms, resulting in the consistent maintenance of liver and renal function parameters. This study has implications in further exploration of the therapeutic potential of active small molecules in BGH to treat DM.

## Figures and Tables

**Table 1:** List of compounds detected in LC-MS.

S. No	Component Name	Formula	Observed m/z	Observed RT (min)
1	Shancilin	C30H28O6	483.1835	0.4
2	5-Hydroxymethyl furoic acid	C6H6O4	141.0194	0.46
3	Isomaltose	C12H22O11	341.1093	0.49
4	Sodium ferulate	C10H9NaO4	215.0318	0.5
5	6″-O-p-Hydroxybenzoyliridin	C31H30O15	641.1532	0.52
6	Raffinose	C18H32O16	503.1622	0.53
7	2-Hydroxy-succinic acid	C4H6O5	133.0145	0.55
8	5R-5-Hydroxymethyl-2(5H)-furanone	C5H6O3	113.0247	0.6
9	Fumaric acid	C4H4O4	115.0039	0.61
10	5,3′,5′-Trihydroxy-6,7,4′-trimethoxy flavone	C18H16O8	359.0756	0.64
11	Iridin	C24H26O13	521.1293	0.64
12	Stachyose	C24H42O21	665.2146	0.66
13	Vanillic acid β-D-glucopyranosyl ester	C14H18O9	329.0886	0.72
14	Succinic acid	C4H6O4	117.0196	1.05
15	Asperuloside	C18H22O11	413.1093	1.52
16	Courmaric acid	C9H8O2	147.0453	2.4
17	Lamiophlomiol A	C11H14O6	241.0719	3.26
18	Geniposidic acid	C16H22O10	373.1141	3.65
19	Leucocyanidin	C15H14O7	305.0666	7.62
20	Gallocatechin	C15H14O7	305.0666	7.62

**Table 2:** All selected compounds showed good binding energies against the active sites of target proteins.

Compound	Binding Energies	
	Amylase	PPAR-Gama
Shancilin	−8.3	−9.7
Iridin	−7.3	−5.5
Stachyose	−6.5	−6.5
Asperuloside	−6.7	−7.4
Gallocatechin	−6.8	−6.3
Lamiophlomiol_A	−6.9	−7.5
Geniposidic_acid	−6.1	−6.2
5,3′,5′-Trihydroxy-6,7,4′-trimethoxy flavones	−6.5	−6.3
Leucocyanidin	−6.5	−6.4
Vanillic_acid_β_D_glucopyranosyl_ester	−6.2	−6.8
Coumaric_acid	−6.7	−7.4
Raffinose	−6.7	−6.2
Isomaltose	−6.4	−5.9
Sodium_ferulate	−5.8	−5.9
Hydroxymethylfuroicacid	−5.2	−5.4
2_Hydroxy_succinicacid	−4.5	−4.9
5_Hydroxymethyl_furanone	−4.7	−4.7
Succinic_acid	−4.3	−4.5
Fumaric_acid	−4.7	−4.7

**Table 3:** Effect of BGH on lipid peroxidation levels, superoxide dismutase activity, catalyse activity, glutathione peroxidase activities in the liver of streptozotocin–nicotinamide-induced diabetic rats.

Parameters	Normal Rats			Diabetic Control	Diabetic Rats		
	Control	1 g/kg/ Day BGH	2 g/kg/ Day BGH		1 g/kg/ Day BGH	2 g/kg/ Day BGH	2 mg/kg/ Day Glibenclamide
Lipid peroxide levels (μ moles of MDA formed/gram wet weight of tissues)	6.27 ± 0.48	5.48 ± 0.59	6.48 ± 0.69	17.38 ± 0.87 *	13.84 ± 0.82 #	12.17 ± 0.97 ##	10.14 ± 0.88 ##
Superoxide dismutase activity (Units/mg protein/min)	3.56 ± 0.09	2.85 ± 0.07	3.25 ± 0.07	0.86 ± 0.08 *	1.26 ± 0.08 #	1.84 ± 0.09 ##	2.06 ± 0.09 ##
Catalyse activity (μ moles of H2O2metabolized/mg protein/min)	2.56 ± 0.06	3.04 ± 0.06	2.89 ± 0.09	0.84 ± 0.05 *	1.20 ± 0.07 #	1.44 ± 0.05 ##	1.65 ± 0.06 ##
Glutathione peroxidase activity (μ moles of GSH consume/mg protein/min)	2.49 ± 0.08	2.32 ± 0.06	2.42 ± 0.06	0.74 ± 0.07 *	1.28 ± 0.06 #	1.42 ± 0.07 ##	1.62 ± 0.09 ##

Values represent means mean ± S.E.M for six rats per group. * *p* < 0.001 compared to normal, non-diabetic rats per group, # *p* < 0.05 compared to diabetic rats group, ## *p* < 0.01 compared to diabetic rats group.

**Table 4:** Effect of BGH on lipid peroxidation levels, superoxide dismutase activity, catalyse activity, glutathione peroxidase activities in the kidney of streptozotocin–nicotinamide-induced diabetic rats.

Parameters	Normal Rats			Diabetic	Diabetic Rats		
		1 g/kg/ Day BGH	2 g/kg/ Day BGH		1 g/kg/ Day BGH	2 g/kg /Day BGH	2 mg/kg/ Day Glibenclamide
Lipid peroxide levels (μ moles of MDA formed/gram wet weight of tissues)	6.97 ± 0.72	6.28 ± 0.87	5.87 ± 0.68	19.48 ± 0.57 *	15.84 ± 0.89 #	12.47 ± 0.97 ##	11.42 ± 0.78 ##
Superoxide dismutase activity (units/mg protein/min)	3.56 ± 0.08	3.65 ± 0.04	3.75 ± 0.09	0.87 ± 0.02 *	1.32 ± 0.12 #	1.74±0.09 ##	1.96 ± 0.13 ##
Catalyse activity (μ moles of H2O2 metabolized/mg protein/min)	2.16 ± 0.07	2.95 ± 0.06	2.49 ± 0.08	0.84 ± 0.02 *	1.08 ± 0.07 #	1.34±0.14 ##	1.65 ± 0.07##
Glutathione peroxidase activity (μ moles of GSH consume/mg protein/min)	2.19 ± 0.11	1.82 ± 0.08	1.97 ± 0.02	0.55 ± 0.04 *	0.87 ± 0.09 #	1.45±0.09 ##	1.62 ± 0.08 ##

Values represent means mean ± S.E.M for six rats per group. * *p* < 0.001 compared to normal, non-diabetic rats group, # *p* < 0.05 compared to diabetic rats group, ## *p* < 0.01 compared to diabetic rats group.

## Data Availability

Not applicable.
